# Cytochrome P450-2D6: A novel biomarker in liver cancer health disparity

**DOI:** 10.1371/journal.pone.0257072

**Published:** 2021-10-01

**Authors:** Zahraa I. Khamis, Xiaodong Pang, Zihan Cui, Qing-Xiang Amy Sang, Jinfeng Zhang

**Affiliations:** 1 Department of Chemistry & Biochemistry, Florida State University, Tallahassee, Florida, United States of America; 2 Laboratory of Cancer Biology and Molecular Immunology, Department of Biochemistry, Faculty of Sciences-I, Lebanese University, Beirut, Lebanon; 3 Insilicom LLC, Tallahassee, Florida, United States of America; 4 Department of Statistics, Florida State University, Tallahassee, Florida, United States of America; 5 Institute of Molecular Biophysics, Florida State University, Tallahassee, Florida, United States of America; King Saud University, SAUDI ARABIA

## Abstract

Liver cancer morbidity and mortality rates differ among ethnic groups. In the United States, the burden of liver cancer in Asian Americans (AS) is higher compared to Caucasian Americans (CA). Research on liver cancer health disparities has mainly focused on environmental and socioeconomic factors yet has ignored the genotypic differences among various racial/ethnic groups. This lack of molecular level understanding has hindered the development of personalized medical approaches for liver cancer treatment. To understand the genetic heterogeneity of liver cancer between AS and CA, we performed a systematic analysis of RNA-seq data of AS and CA patients from The Cancer Genome Atlas (TCGA). We used four differential gene expression analysis packages; DESeq2, limma, edgeR, and Superdelta2, to identify the differentially expressed genes. Our analysis identified cytochrome P450-2D6 enzyme (CYP2D6) as the gene with the greatest differential expression with higher levels in AS compared to CA. To scrutinize the underlying mechanism of CYP2D6, Ingenuity Pathway Analysis (IPA) and Cytoscape were conducted and found hepatocyte nuclear factor-4α (HNF4A) and interleukin-6 (IL6) in direct association with CYP2D6. IL6 is downregulated in AS compared to CA, while HNF4A is not significantly different. Herein, we report that CYP2D6 may serve as a putative biomarker in liver cancer health disparities. Its negative association with IL6 proclaims an intricate relationship between CYP2D6 and inflammation in the ethnic differences seen in AS and CA liver cancer patients. The goal of the present study was to understand how genetic factors may contribute to the interethnic variability of liver cancer prevalence and outcomes in AS and CA patients. Identifying ethnic-specific genes may help ameliorate detection, diagnosis, surveillance, and treatments of liver cancer, as well as reduce disease-related incidence and mortality rates in the vulnerable population.

## Introduction

Liver cancer is a major global health problem and a leading cause of death worldwide. The World Health Organization estimates that more than 1 million deaths will occur from liver cancer in 2030 [1]. In 2021, it has been estimated that 42,230 people will be diagnosed with liver cancer and 30,230 will die from the disease in the United States [2]. Liver cancer incidence continues to rise in 2020 at a faster pace compared to other cancers. The major etiologic factors for this cancer include metabolic disorders like diabetes and obesity, chronic infection with hepatitis B or C viruses, smoking, and excessive alcohol consumption [3]. Despite these seemingly preventable risk factors, the 5-year survival rate of liver cancer is only 18% in 2020, ranking it the second most fatal tumor after pancreatic cancer [2].

Disparities in liver cancer incidence and mortality rates are reported among populations with different races and ethnicities. Latinos have suffered from the highest increase in liver cancer incidence in the past years. Compared to Caucasian patients with different etiologies, Latinos presented with more advanced and severe features of the disease [4]. Other factors contributing to disparities include age, gender, socioeconomic status, access to health care, and geographic location. A mounting evidence suggests that genetic and epigenetic factors can predict an individual’s susceptibility to developing liver cancer. In a Caucasian population, five genetic biomarkers have been identified as significant determinants of liver cancer risk [5]. Liver cancer disproportionally affects Asians and Caucasians in the United States with higher incidence among Asian groups [6]. This disparity has never been explored at the genetic level between the aforementioned groups. So, we opt to identify the genes differentially expressed in AS and CA liver cancer patients to improve disease screening, early cancer detection, and therapies tailored to individual’s needs.

**Fig 1:**
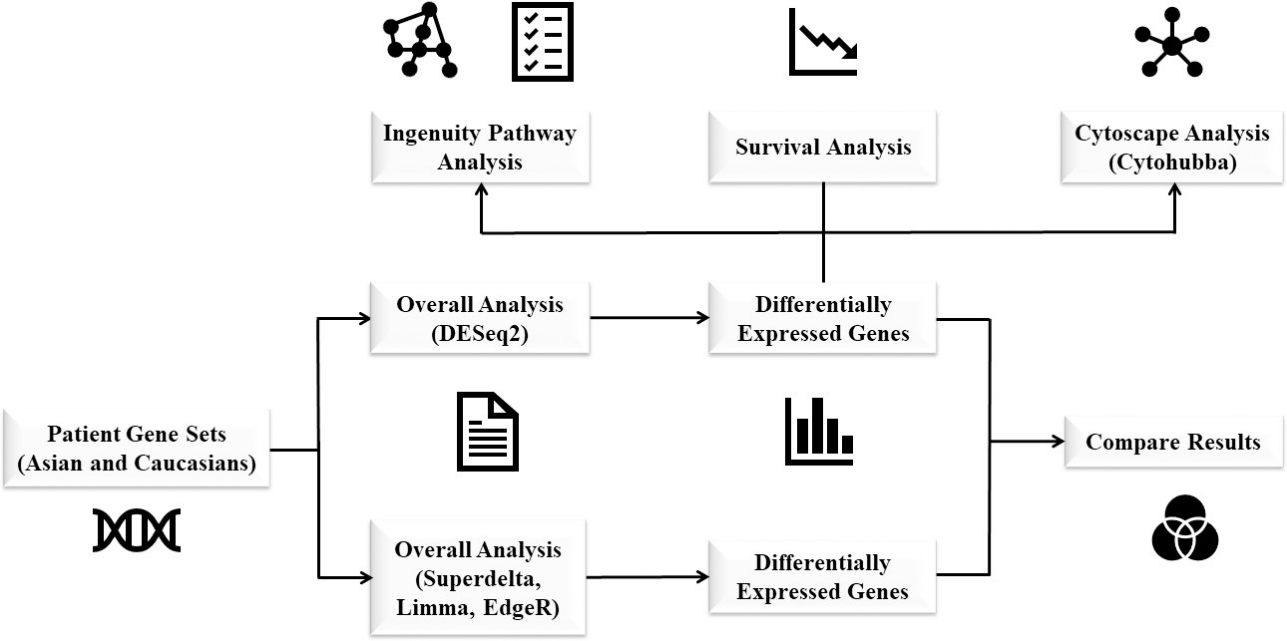
Work flowchart used for data analysis.

To determine differentially expressed genes (DEGs), we have performed an integrative computational analysis of gene expression data from The Cancer Genome Atlas (TCGA) of AS and CA liver cancer patients. The use of four different R/Bioconductor packages: DESeq2 [7], limma [8], edgeR [9], and Superdelta2 [10] maximized the statistical power of our study. We have found 19 common DEGs among the four packages with CYP2D6 on the top of the list. CYP2D6 exhibited increased expression in AS compared to CA. To gain more insights about the underlying molecular pathways associated with CYP2D6, IPA and Cytoscape plug-in, Cytohubba, were used to generate integrated pathways, networks, and upstream regulators, and to identify important hub genes, respectively [11]. Using these analyses, we have identified key genes that may decrease ethnic-driven health disparities and may serve as valuable prognostic and therapeutic targets that can stop the development of liver cancer (Fig 1).

## Materials and methods

### RNA-seq data from TCGA

The RNA-seq data of all liver hepatocellular carcinoma (LIHC) tumor samples of AS (159) and CA (185) were downloaded from The Cancer Genome Atlas (TCGA) (http://cancergenome.nih.gov/). Normalized gene expression counts were used, unless otherwise noted. The complete list of patient IDs is found in S1 Table.

### Differential gene expression analysis

To calculate the DEGs between AS and CA, with CA as the reference group, four different methods were used: DESeq2 (version 1.30) [7], limma (version 3.46.0) [8], edgeR (version 3.32.0) [9], and Superdelta2 (version 2.0) [10]. For each method, genes with the log_2_ fold-change greater than 1 and adjusted p-value smaller than 0.05 were selected as DEGs. Genes with a sum of 10 reads or fewer across all samples were removed before analysis. The Benjamini-Hochberg method was used for p-value adjustment [12]. For DESeq2, parametric fit type was used. Outliers replacement, outliers filtering, and independent filtering were on. For limma, RNA-seq data were first transformed with the voom function. The robust settings were applied while the test significance relative to a fold-change threshold was not. For edgeR, the robust settings were applied. For Superdelta2, offset of 0.5 was added to avoid infinite value of log_2_ of 0. The trimming portion was set to 0.2 when estimating the between-group test. The heatmap was generated using R package pheatmap (version 1.0.12).

### Pathway and network analysis

The DEGs from DESeq2 were submitted to the IPA software to determine the relative enrichment of genes in different pathways using right-tailed Fisher’s exact test. Based on the differential expression data, IPA analysis generated canonical pathways, upstream regulators, and top tox list. Hub genes were identified using the CytoHubba plug-in in the Cytoscape software.

### Survival analysis

Survival analysis was performed using the survival (version 3.2–7) and survminer (version 0.4.8) packages in R.

## Results

### Genotypic profiling identifies 19 common differentially expressed genes

**Fig 2:**
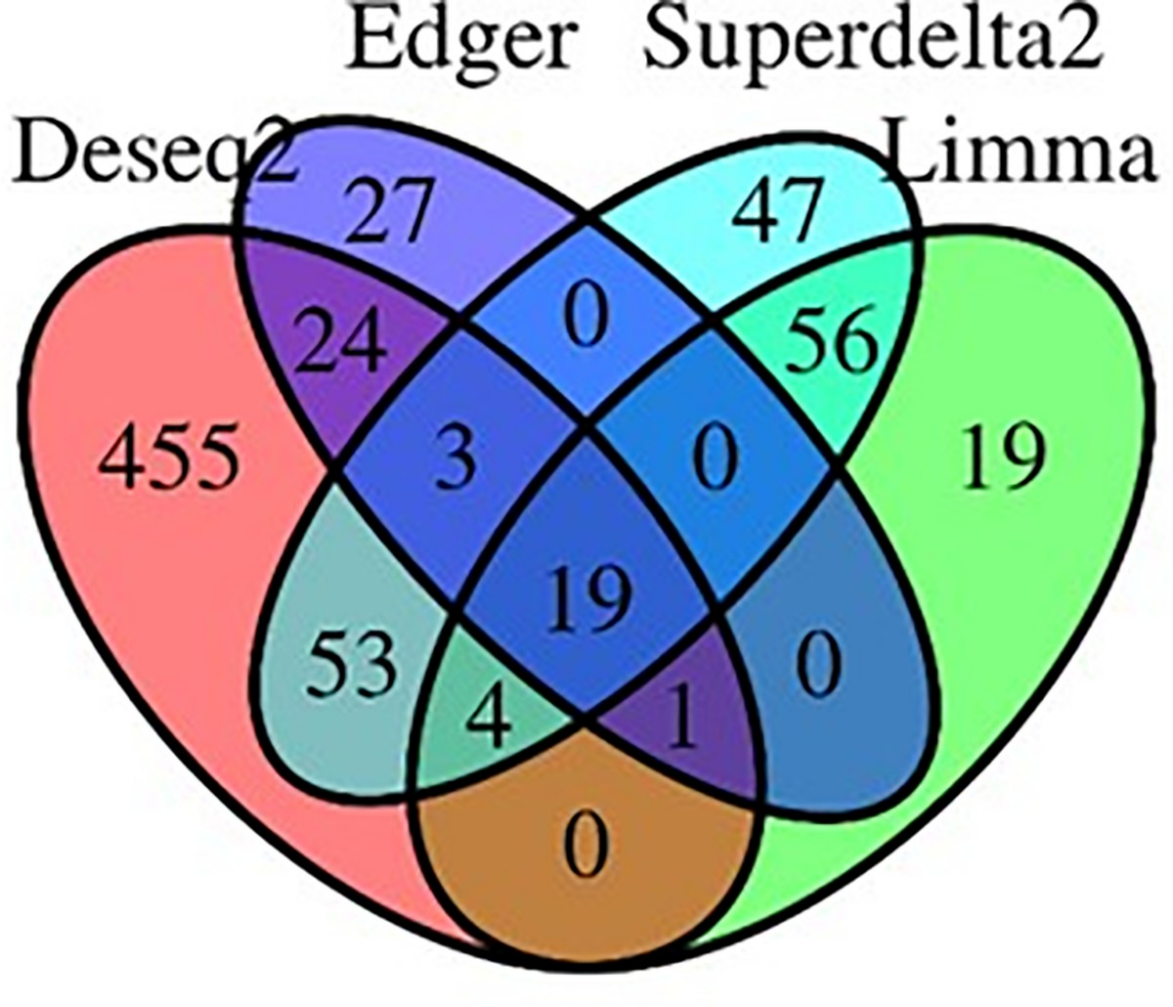
Venn diagram of DEGs. Four different methods (DESeq2, edgeR, limma, and Superdelta2) were utilized generating 19 common differentially expressed genes.

To uncover the genes that may contribute to disparities in liver cancer incidences between AS and CA, we used several analysis methods—DESeq2 [7], limma [8], edgeR [9], and Superdelta2 [10]—to identify the differentially expressed genes. An overlapping set of genes representing the strongest contributors to the disparities in liver cancer incidences was determined. For each method, we selected the significant genes with an absolute log_2_ fold-change >1 (log_2_ FC> 1) and adjusted p-value <0.05 as differentially expressed. The DESeq2 package revealed a set of 559 differentially expressed genes between AS and CA patients. Analysis from limma, edgeR, and Superdelta2 pipelines revealed 99, 74, and 182 differentially expressed genes, respectively (S2 Table). Only 19 differentially expressed genes are common among the four methods (Fig 2). Table 1 lists the details of these genes. A negative log_2_ FC reveals a downregulation in AS compared to CA and a positive log_2_ FC shows an upregulation in AS compared to CA. Out of the 19 common DEGs, 6 genes are upregulated and 13 genes are downregulated.

**Table 1:** The common genes with differential expression.

Gene ID	log_2_ FC	p-value	padj
CYP2D6	1.82	8.67e-27	5.66e-23
SLC39A4	1.68	5.95e-14	4.22e-11
COL9A3	1.24	6.17e-9	7.49e-7
TSPAN10	1.2	7.58e-18	1.48e-14
DHRS2	1.18	0.0000429	0.000748
ACSM1	1.11	0.00000647	0.000176
GREM2	-1.01	0.000259	0.00284
ESR1	-1.01	0.00000566	0.000159
GSTA2	-1.03	0.0000469	0.000798
TAT	-1.03	0.0000219	0.00045
ADH1B	-1.03	5.77e-7	0.0000302
PPM1K	-1.04	1.4e-14	1.25e-11
SRD5A2	-1.1	0.00000495	0.000144
CFHR4	-1.11	5.09e-7	0.0000272
CPED1	-1.14	9.39e-12	3.67e-9
MFAP3L	-1.17	2.5e-10	5.32e-8
CLRN3	-1.28	0.00000105	0.000046
GNAO1	-1.38	2.16e-10	4.75e-8
SPATA18	-1.48	4.66e-13	2.34e-10

The list of the 19 DEGs that are common among the four analysis methods used. The genes are listed with their respective fold change (log_2_ FC), the p-value, and the adjusted p-value (padj).

### CYP2D6 as a potential biomarker in Asian Americans with liver cancer

**Fig 3:**
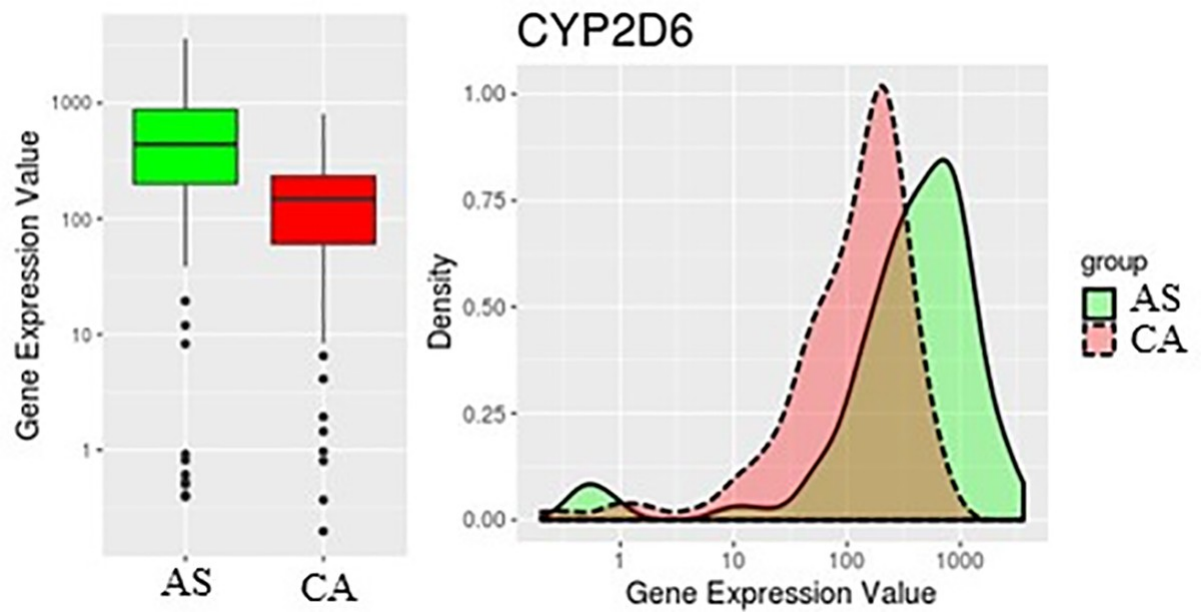
Expression levels of CYP2D6 in AS and CA groups. CYP2D6 is higher in AS than CA.

Table 1 shows CYP2D6 as the highest significantly differentially expressed gene in our data set. The CYP2D6 (Cytochrome P450 Family 2 Subfamily D Member 6) gene encodes a member of the cytochrome P450 superfamily of enzymes and is involved in the metabolism of drugs and other endogenous and exogenous molecules [13]. CYP2D6 (log_2_ FC = 1.82) is overexpressed in AS compared to CA (Fig 3). This is in agreement with our previous report about breast cancer health disparities where AS breast cancer patients experience higher levels of CYP2D6 compared to their CA counterparts [14].

### Ingenuity pathway analysis identifies HNF4A as an upstream regulator of CYP2D6

**Fig 4:**
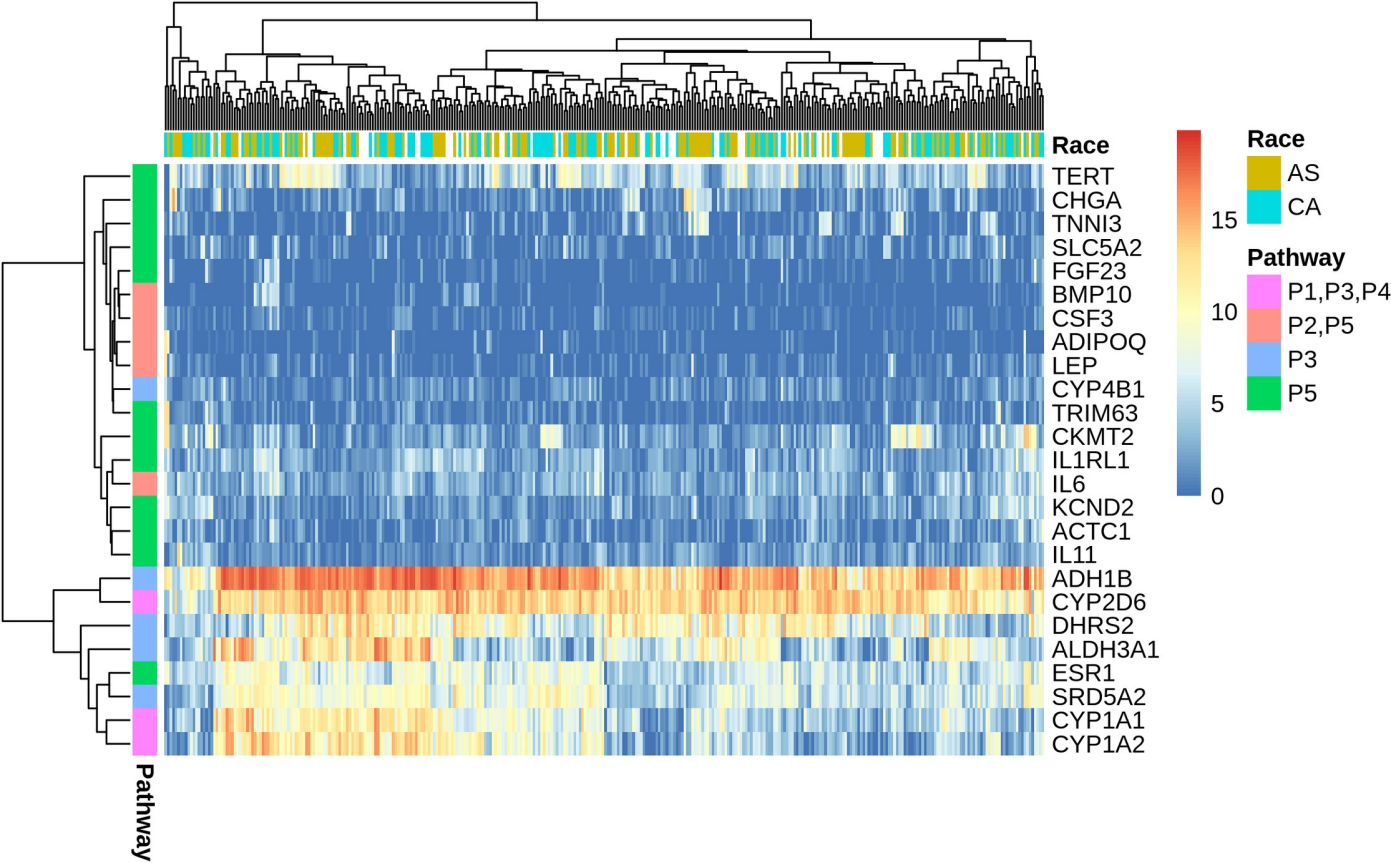
Heatmap of log_2_ FC value of differentially expressed genes in the top 5 tox pathways. CYP2D6 has high expression and close association with P1, P3, and P4 pathways pertinent to its normal biological functions in xenobiotics and fatty acid metabolism.

**Fig 5:**
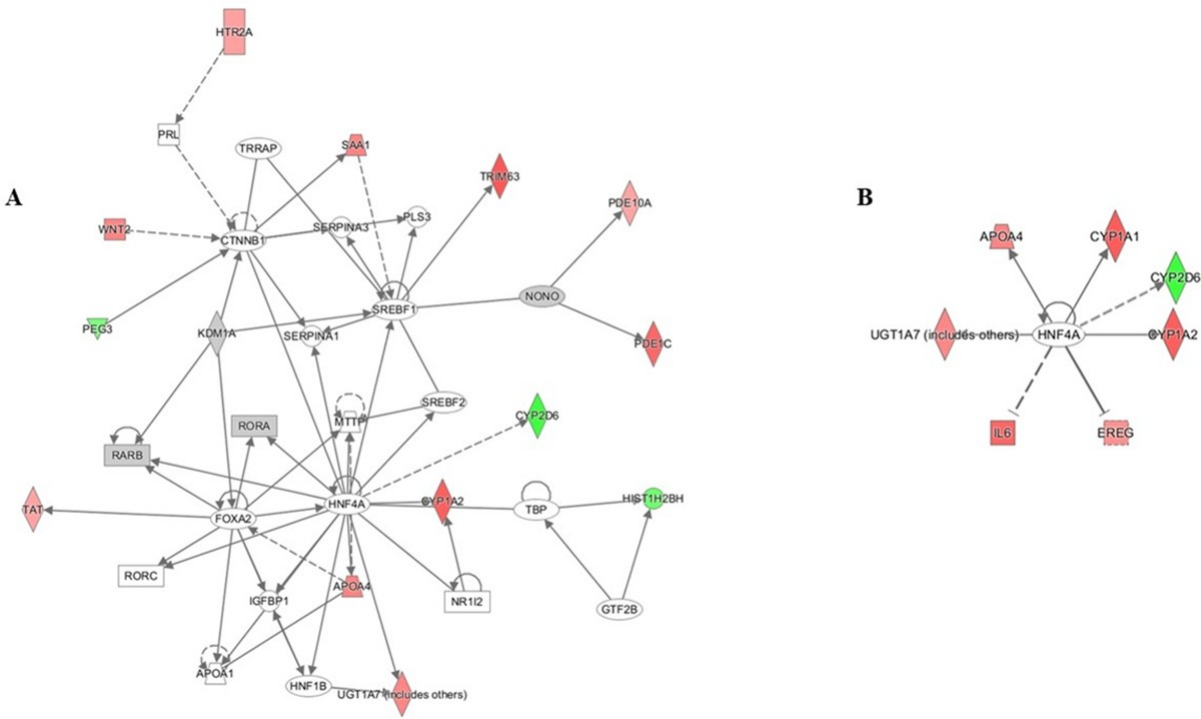
IPA analysis of differentially expressed genes. (A) Network analysis reveals a direct interaction between CYP2D6 and HNF4A. (B) Upstream network analysis of HNF4A shows CYP2D6 and IL6 as major players.

To decipher the role of CYP2D6 in the disparity of liver cancer incidence and outcome between AS and CA, we imported the DESeq2 differentially expressed dataset of 559 genes into the Ingenuity Pathway Analysis (IPA) tool. IPA uses the Ingenuity Knowledge Database to map DEGs into networks, canonical pathways, and a top tox list to determine functional relationships. The IPA tox list analyses revealed a significant enrichment of cytochromes P450 enzymes, xenobiotic metabolism, and fatty acid metabolism. Table 2 displays the most significantly altered pathways in our dataset based on the tox list tool analysis. Heatmap of DEGs in the top five tox pathways shows our gene of interest, CYP2D6, to be highly associated with P1, P3, and P4 pathways which directly correlates with CYP2D6 function in fatty acid metabolism and xenobiotics (Fig 4). Additional IPA network analysis of CYP2D6 shows its association with HNF4A (Fig 5A). HNF4A is a highly expressed receptor in the liver and is known to regulate liver development and metabolic functions [15, 16]. To gain more biological insight into the role of HNF4A, an upstream regulator analysis was performed. IPA depends on experimentally-observed biological findings available in the literature to predict upstream regulatory molecules based on input genes [17]. Interestingly, our analysis identified HNF4A as an upstream regulator of the DEGs in our dataset. Therefore, this gene may contribute to the observed gene expression changes seen in AS and CA liver cancer patients in this study.

**Table 2:** The top toxicological pathways.

Name	Pathway	P-value
P1	Cytochrome P450 panel- substrate is a xenobiotic (human)	9.05 E-04
P2	Increases cardiac proliferation	1.36 E-03
P3	Fatty acid metabolism	3.25 E-03
P4	Cytochrome P450 panel- substrate is a xenobiotic (Rat)	3.72 E-03
P5	Cardiac hypertrophy	1.01 E-03

The pathways are predicted by Ingenuity Pathway Analysis tool based on the differentially expressed genes taken from DESeq2 pipeline.

### Cytohubba analysis identifies IL6 as a hub gene among DEGs in liver cancer

**Fig 6:**
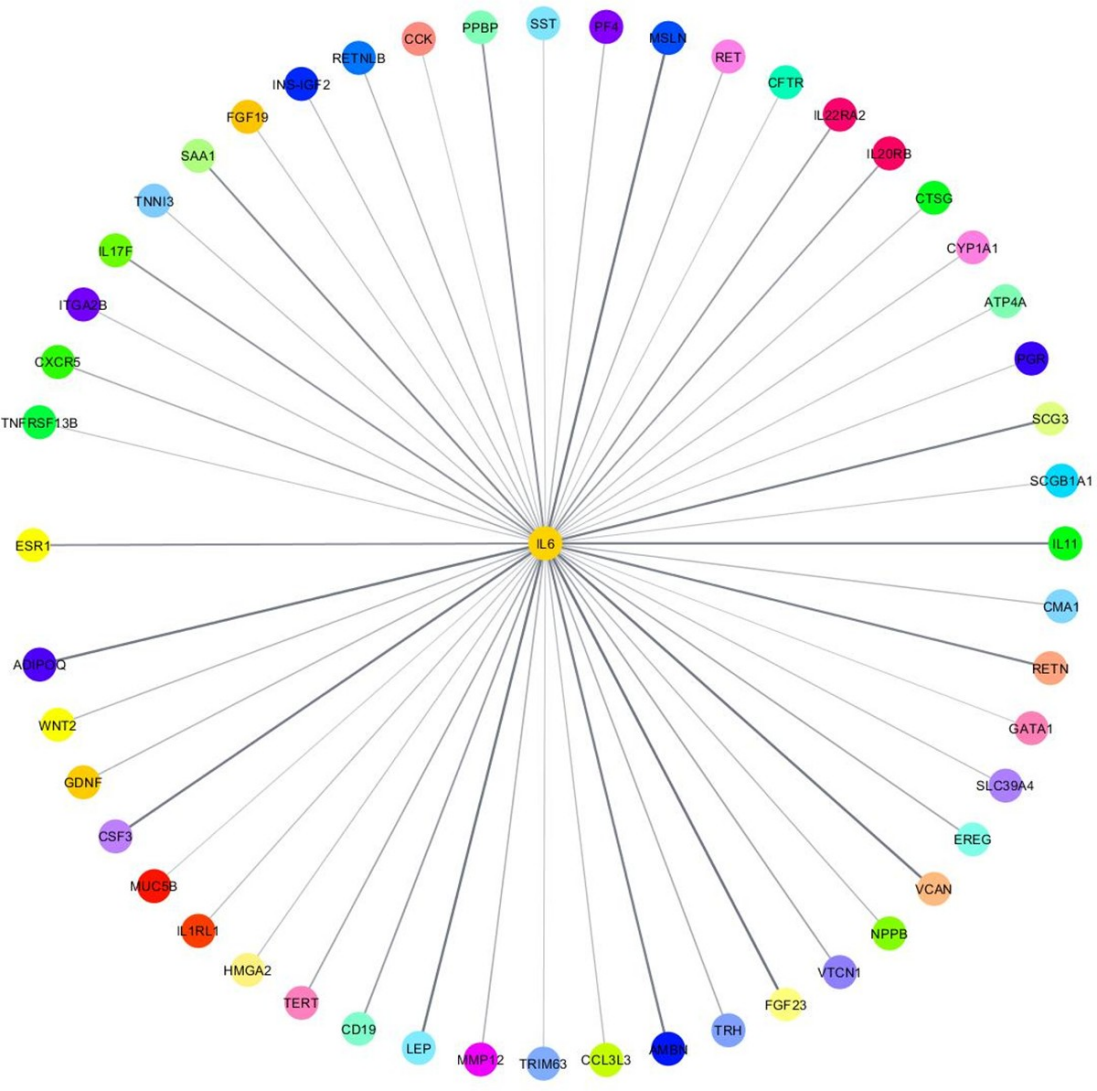
IL6 as a hub gene. IL6 has the highest degree of connectivity.

In search for the underlying mechanism that may explain the disparity between AS and CA liver cancer patients, we have performed an IPA network analysis on the upstream regulator, HNF4A. The interacting molecules include enzymes (CYP1A1, CYP1A2, CYP2D6, UGT1A7), a growth factor (EREG), a cytokine (IL6), and a transporter (APOA4) (Fig 5B) (Table 3). Based on previous reports, IL6 cytokine expression is inversely correlated with the activity of cytochrome P450 drug metabolizing enzymes [18], the category in which the most significant DEG, CYP2D6, falls. Thus, IL6 is further pursued. Cytoscape analysis of our DESeq2 differentially expressed genes using cytohubba plugin has revealed IL6 as the central node with the highest degree of connectivity (Fig 6). Therefore, the highly connected IL6 node is an important biological hub gene in our dataset (Table 4). IL6 is downregulated in AS compared to CA liver cancer patients (log_2_ FC = -1.72). Shi et al. has reported a similar decrease in IL6 expression in AS breast cancer patients compared to their CA counterparts supporting our results [19]. Hence, IL6 and CYP2D6 expression levels are inversely proportional in accordance with the known effects of IL6 on cytochrome P450 enzymes [18].

**Table 3:** Upstream network analysis of HNF4A.

Symbol	Entrez Gene Name	Log Ratio	p-value
APOA4	apolipoprotein A4	-1.37	0.0000648
CYP1A1	cytochrome P450 family 1 subfamily A member 1	-1.83	3.26E-08
CYP1A2	cytochrome P450 family 1 subfamily A member 2	-1.8	0.000000922
CYP2D6	cytochrome P450 family 1 subfamily D member 6	1.82	8.67E-27
EREG	Epiregulin	-1.23	0.000397
IL6	interleukin 6	-1.72	6.05E-14
UGT1A7	UDP glucuronosyltransferase family 1 member A	-1.35	0.00406

The molecules correlated with HNF4A when network analysis upstream of HNF4A is performed using IPA. Expression levels and p-values are shown.

**Table 4:** Top 10 essential genes in our DEGs dataset.

Rank	Name	Score
1	IL6	50
2	SST	33
3	CCK	28
4	SAA1	27
5	PVALB	25
6	GRIN1	25
7	GPR17	24
8	LEP	23
9	ADCY1	22
10	LPAR3	22

Cytoscape analysis using Cytohubba-plugin reveals IL6 as the highest interconnected gene based on the degree method.

### Survival analysis

**Fig 7:**
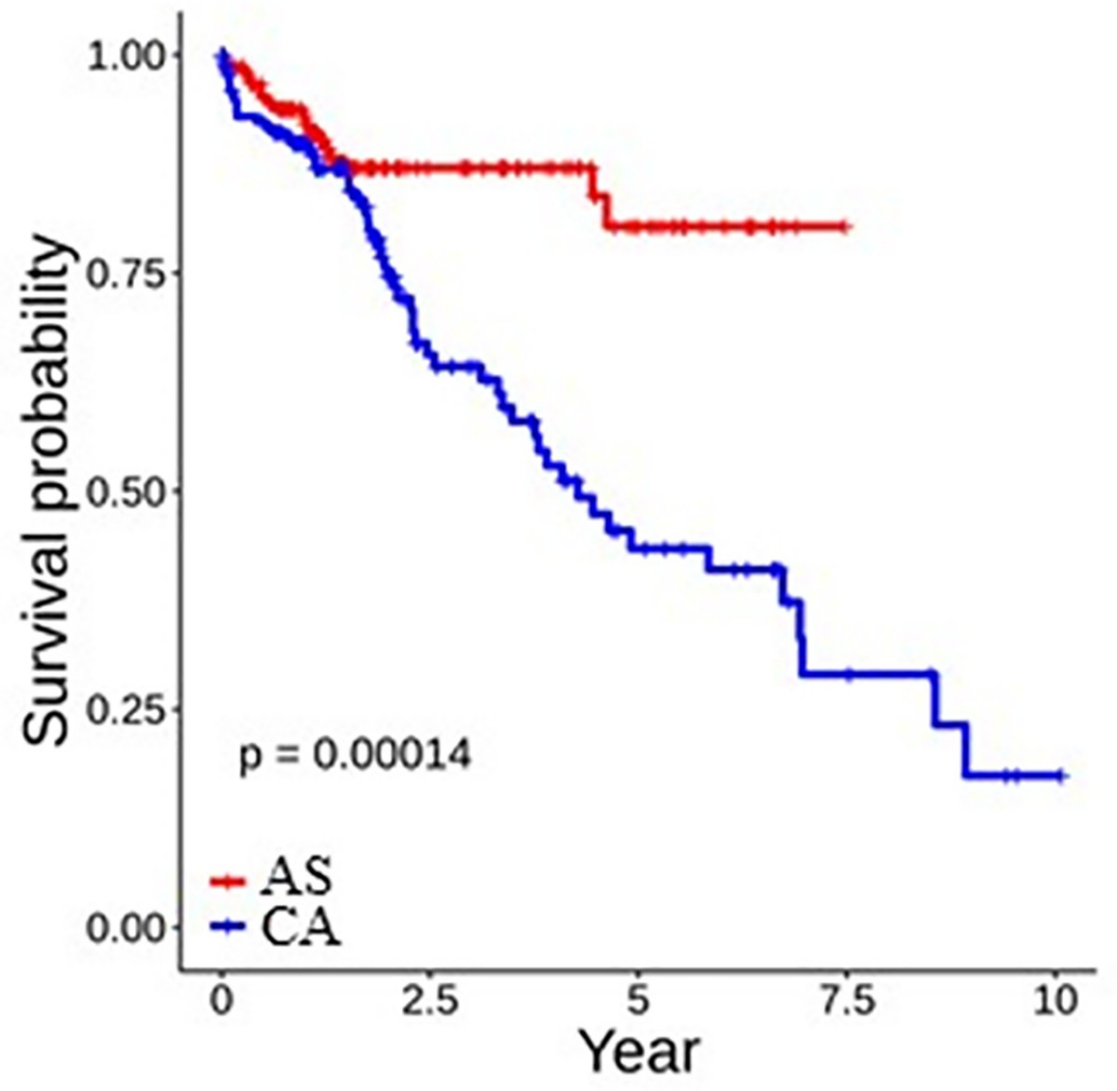
Survival curve of AS and CA groups. AS have higher survival rate that CA.

Survival analysis indicates significant differences in survival between AS and CA (p-value = 0.00014) (Fig 7). Although they have a greater liver cancer incidence rate than CA, AS patients display a much higher 5-year survival rate of ~80% compared to CA, which is ~45%.

## Discussion

Liver cancer is a leading cause of cancer-related deaths worldwide. Like all types of cancer, liver cancer incidence, prevalence, and mortality rates vary substantially between different populations [20]. These health disparities are caused by a combination of tumor biology, environmental, social, and economic factors. The contribution of the genotypic differences among various populations is underexplored [21, 22]. In a retrospective study of racial and ethnic minorities diagnosed with liver cancer, Rich et al. have reported that variations in liver cancer outcomes are associated with delays in diagnosis and receipt of curative treatment [23]. Additionally, evaluation of liver cancer incidence by Yang et al. in California based on race/ethnicity, socioeconomic status, and geographic area has found that Asians/Pacific Islanders (APIs) and Hispanics have the highest incidence rate [24]. Among US populations, Asians have a higher liver cancer burden than other racial groups. However, in our study, we have found that AS have a much higher survival rate than CA (Fig 7). The etiology of this disparity has never been investigated at the genetic level. Therefore, we have examined the genotypic differences between AS and CA patients to determine the genetic basis of the racial/ethnic specific patterns of liver cancer incidence. To our knowledge, this is the first study to identify differentially expressed genes in AS and CA that may potentially drive the observed health disparity.

In this study, we analyzed differentially expressed genes (DEGs) in AS versus CA liver cancer tumor samples. To make the results more robust, we used four differential expression analysis packages, DESeq2, limma, edgeR, and Superdelta2, and identified 19 common DEGs (Fig 2 and Table 1). The major drug-metabolizing enzyme, CYP2D6, displayed the highest significant change (log_2_ FC = 1.82) (Fig 3). This gene is expressed in various tissues, including those of the liver, kidney, intestine, and breast [25]. Although it constitutes ~1.5% of total hepatic CYP enzymes, CYP2D6 metabolizes ~30% of commonly used drugs [26]. Located on chromosome 22q13, CYP2D6 gene is highly polymorphic, with more than 90 described allelic variants. These variants—mainly due to single nucleotide polymorphisms (SNPs), gene deletions, or gene duplications—usually result in abolished or altered activity of the CYP2D6 enzyme [13]. Depending on the function of each allele in CYP2D6 gene, the enzyme can have four different phenotypes; poor metabolizer (PM), intermediate metabolizer (IM), extensive metabolizer (EM), and ultra-rapid metabolizer (UM) [27]. The distribution of the allelic variants shows a substantial interindividual differences. Caucasians and African Americans populations have higher PM frequency than the Chinese population highlighting the influence of CYP2D6 genotypic variation on its phenotype and thus, its expression and function [28]. The genetic polymorphism of CYP2D6 in various populations, together with its role in the metabolism of a large percentage of xenobiotics and administered drugs should be taken into consideration in clinical settings while assessing the therapeutic and toxic dose response of medications prescribed for patients.

The relationship between CYP2D6 allelic variants and cancer incidence is still debatable due to conflicting reports [29, 30]. CYP2D6 has been extensively studied in breast cancer due to its ability to convert tamoxifen to the more active metabolite, endoxifen [31]. Patients with poor metabolizer phenotype for CYP2D6 show lower concentration of endoxifen [27]. With respect to the liver, few reports have claimed a significant association between CYP2D6 polymorphism and the development of liver cancer. One study has showed that individuals who are homozygous for functional CYP2D6 alleles are at an increased risk of developing the disease [32, 33]. Also, individuals with hepatitis C virus (HCV) are reported to have lower activity of CYP2D6 in the liver [34]. However, assessment of CYP2D6 in liver cancer patients with different racial/ethnic backgrounds has not been reported.

Given CYP2D6 significant role in drug clearance, evaluation of CYP2D6 in liver cancer patients of diverse backgrounds must be prioritized to ensure the proper application of pharmacotherapeutics in precision medicine. In our analysis, CYP2D6 shows the greatest significant differential expression in the more vulnerable population. It has increased expression levels in AS than CA liver cancer patients (Fig 3), which is in accordance with our previous findings in breast cancer [14]. Therefore, CYP2D6 may serve as a putative biomarker in liver cancer health disparities. To understand the role of this gene in our dataset, we performed an IPA functional analysis. The top tox list tool of IPA identified the most significant pathways and gene lists in our input data to be cytochrome P450 enzymes, xenobiotic metabolism, and fatty acid metabolism (Table 2). Heatmap generation of DEGs in these pathways revealed high expression and clustering of CYP2D6 to these pathways, consistent with its biological roles in liver metabolism (Fig 4).

Additional IPA network analysis reveals that HNF4A acts as a regulator of CYP2D6 expression (Fig 5A). HNF4A is a member of the nuclear receptor superfamily that binds DNA elements and regulates gene transcription. Mainly expressed in the liver, it is essential for the proper maintenance of hepatic development and metabolic functions. Mice that are unable to express HNF4A have altered lipid metabolism and increased mortality, which underscores the key regulatory role of HNF4A in liver physiology [16, 35, 36]. Studies indicate that HNF4A is indeed a regulator of CYP2D6 [37] with HNF4A expression and CYP2D6 enzyme activity significantly correlated [38, 39]. Although the expression of HNF4A was not significantly different between AS and CA liver cancer patients in our study, our IPA analysis did identify HNF4A as an upstream regulator of CYP2D6.

In the HNF4A upstream regulator network, a set of DEGs are in direct association with HNF4A (Fig 5B); most importantly, interleukin-6 (IL6), a known effector of cytochrome P450 enzymes. IL6 is a pleiotropic cytokine that acts through its receptor (IL6R) to elicit a wide variety of effects ranging from acute phase response (APR) to proliferation, differentiation, and other biological processes [40, 41]. It is negatively associated with cytochrome P450 (CYP450) enzyme activity and HNF4A expression [18, 42]. In vitro exposure of human hepatocytes in cell culture to IL6 caused suppression of CYP450 enzymes [18]. The IL6-mediated suppression of liver CYP450 enzymes has been an issue in the drug industry because most prescribed drugs are CYP-metabolized. So, efforts have been made to block this cytokine using an anti-IL6 receptor antibody to reduce the bioavailability of CYP-metabolized medications [18, 43]. With respect to CYP2D6, primary cultures of human hepatocytes treated with IL6 caused a time- and dose-dependent decrease in CYP2D6 expression [44]. Other proinflammatory cytokines like tumor necrosis factor α (TNFα) and interleukin 1β (IL1β) increased as the nonalcoholic fatty liver disease progressed. This increase in TNFα and IL1β levels was accompanied with a decrease in CYP enzymes, among which was CYP2D6. The authors implied that the elevated expression in cytokines may have caused the observed decrease in CYP enzymes activity [45]. The drug metabolizing CYP450 enzymes are known to induce or inhibit inflammation based on their ability to metabolize a variety of molecules. The role of CYP450 enzymes in inflammation is reviewed extensively by Christmas P. [46]. Our finding, which shows an inverse expression of IL6 and CYP2D6, is consistent with the literature. IL6, with a log_2_ FC = -1.72, is downregulated in AS compared to CA. The importance of IL6 in our liver cancer health disparity dataset was further validated using Cytohubba plug-in, which identified IL6 as a hub gene with the highest degree of connectivity (Fig 6). Collectively, these results may indicate a relationship between inflammation and CYP enzymes; in particular, CYP2D6.

Based on their role in the metabolism of therapeutic drugs, xenobiotics, and endogenous substrates, CYP450 enzymes play major roles in inflammation and cancer. Generally, inflammation decreases the expression and activity of CYP450 enzymes due to the release of proinflammatory cytokines from immune cells. In the context of tumor microenvironment, the modulation of CYP450 levels in the presence of proinflammatory cytokines can induce tumorigenesis and affect response to chemotherapy [47]. Thus, it is indispensable to elaborate the inflammation-CYP450-cancer axis to determine drug-drug interactions and proper drug dosages to avoid adverse effects and therapeutic failure. Our results positions CYP2D6 at the intersection between inflammation and liver cancer in AS and CA patients. The high levels of CYP2D6 in AS and IL6 in CA suggest variability in liver cancer etiology. Elevated IL6 expression in CA patients may imply an initial hepatic inflammation due to HBV/HCV that may have progressed to liver cancer; while the increased levels of CYP2D6 in AS may be due to another underlying problem like alcohol or metabolic syndrome. Therefore, it is of paramount importance to delineate the predominant cause of liver cancer in distinct populations, and to investigate how the underlying cause can affect the expression and activity of CYP2D6, and the release of proinflammatory cytokines, mainly IL6.

Our findings suggest an intricate interplay between the genes CYP2D6, HNF4A, and IL6 in AS and CA liver cancer patients. Research in the past decade has shown that these three genes are highly polymorphic, leading to variability in the function of the respective gene products. In a Korean population, the genetic polymorphism G60D in HNF4A resulted in low activity of CYP2D6 [48]. Another study in *Schistosoma haematobium*-infected patients from Africa found that certain polymorphic variants of CYP2D6 and IL6 were highly associated with morbidity and severity of the disease [49]. Thus, we contemplated that the relationship between CYP2D6, HNF4A, and IL6 was governed by single nucleotide polymorphisms (SNPs), specifically in CYP2D6. The availability of different allelic variants in each locus may dictate how each gene will interact with the others and regulate the observed disparity in AS and CA liver cancer patients. Surprisingly, SNP analysis revealed only one variant (ID: rs72657357) associated with IL6 expression with a frequency of 0.0239 in CA and 0.0179 AS (S3 Table). Neither CYP2D6 nor HNF4A had SNPs related to the health disparity between the two ethnic groups obviating the implication of CYP2D6 polymorphism in the inequalities of liver cancer in AS and CA. Therefore, it is the gene expression level of CYP2D6 that contributes to the liver cancer health disparity.

Liver cancer prevalence in AS is higher than any racial/ethnic population. However, survival analysis of our dataset showed a significant difference in survival between AS and CA patients. AS had a prolonged 5-year survival rate of 80%, while CA had a 45% rate (Fig 7). This result suggests that increased CYP2D6 expression levels are associated with better liver cancer survival rate; however, multiple biological, environmental, social, economic, cultural, and other confounding variables associated with liver cancer survival remain to be investigated.

## Conclusions

The dismal prognosis of liver cancer necessitates the development of new therapeutics that can target all cancer patients regardless of their differences. With prevalence of liver cancer health disparities, efforts are tailored to individualize disease origin, diagnosis, and treatment for each patient to optimize better responses to therapy. To eliminate health disparities, we need to understand how society, environment, lifestyle, and ancestral factors affect liver cancer incidence and outcome. This is initiated by a comprehensive profiling of the genome. Herein, we explored how genetic differences in AS and CA patients might influence liver cancer incidence and survival. Being the highest significantly DEG among the two populations, CYP2D6 may serve as a putative biomarker for liver cancer disparities. The higher expression of CYP2D6 in AS can be attributed to higher disease risk, better survival outcome, or different etiology profiles. Deciphering how CYP2D6 affects incidence or survival rates warrants further investigation. It is also imperative to dissect the inherent causes of liver cancer in AS and CA, and their effect on CYP2D6 activity and inflammatory responses. Once elucidated, we will have a better understanding of the way CYP2D6 modulates liver cancer and inflammation; thus, leading to better assessment of drugs interactions and conduct of efficient therapies with minimal side effects. Although these preliminary results require further validation, CYP2D6 and IL6 might have significant clinical impact in the management of liver cancer incidence and application of personalized treatment regimens.

## Supporting information

S1 Table Patient IDs and clinical information. (XLSX)

S2 Table Differentially expressed genes in AS and CA liver cancer patients. Results from all methods (DESeq2, edgeR, limma, and Superdelta2) are displayed.
(XLSX)

S3 Table Single nucleotide polymorphism analysis. SNPs identified in AS and CA liver cancer patients.
(XLSX)
